# Teaching and Curriculum of the Preschool Physical Education Major Direction in Colleges and Universities under Virtual Reality Technology

**DOI:** 10.1155/2022/3250986

**Published:** 2022-03-09

**Authors:** Nina Wang, Mohd Nazri Abdul Rahman, Boon-Hooi Lim

**Affiliations:** ^1^Department of Educational Psychology & Counselling, Faculty of Education, University of Malaya, 50603 Kuala Lumpur, Malaysia; ^2^Centre for Sport and Exercise Sciences, University of Malaya, Kuala Lumpur 50603, Malaysia

## Abstract

The present work aims to study the influence of virtual reality (VR) technology on the teaching and curriculum of preschool physical education in colleges and universities and establish a virtual teaching model suitable for the college teaching system. The classroom teaching situation of using VR technology in physical training of preschool education major direction in colleges and universities is investigated using the questionnaire survey and teaching experiment. Firstly, the feasibility of applying VR technology to teaching is proved by analyzing the relevant education theories. Secondly, the experimental research method is designed to verify the application effect of VR technology in teaching behavior. Finally, the collected data is sorted out to judge the method's feasibility. The experimental results demonstrate that 88.0% of the respondents are curious about the application of VR, and 88.6% of the respondents can accept the application of VR in sports dance teaching. Besides, 89.1% claimed that VR technology could enhance students' understanding of knowledge, and 93.0% thought that VR applications would not interfere with teachers' explanations. In addition, 80.0% thought that virtual teaching could stimulate students' interest in learning, 75.0% said that VR application could attract students' attention, and 63.0% believed that VR application could improve learning efficiency. The preliminary investigation suggests that introducing VR technology can reduce the work intensity of teachers, and the students participating in the survey are optimistic about the application of VR technology in preschool physical education. The formal test based on a *t*-test indicates that the average score of the experimental group is 3.18 points higher than that of the control group, and there are significant differences in technology between the control group and the experimental group. VR technology can improve students' technical level, enhance self-confidence, and improve students' grades. To sum up, this study provides a reference for developing virtual teaching mode and applying VR technology to physical education.

## 1. Introduction

Textbooks and blackboards are standard traditional teaching aids in colleges and universities and the basis of teaching [1, 2]. Education and teaching activities are information exchange processes between students and teachers mediated by certain media. Utilizing multimedia tools such as video, image, and animation in the classroom optimizes the teaching process and enriches the learning materials. However, the traditional teaching model and curriculum influence these novel teaching methods, impede multimedia and other modern equipment in the classroom, and hinder students' subjective initiative. Generally speaking, it is easy for multimedia technology to cause students to be in a passive state and teachers to rely too much on multimedia courseware. Therefore, the inevitable teaching development is promoting the transformation of teaching methods and curriculum, from multimedia to two-dimensional and three-dimensional education, and finally to virtual teaching.

Virtual reality (VR) technology first rose in the 1960s, covering many technical categories such as image, multimedia, and simulation technology, which can construct a computer simulation system. Therefore, VR technology has been applied in many fields [3, 4]. In terms of composition, VR technology contains computer processing and mutual transformation between physical objects and virtual objects [5]. VR technology involves various technical methods such as sound positioning, model construction, and spatial tracking. VR technology can give play to its outstanding advantages through integrating multiple technologies and methods [6]. Immersion, human-computer interaction, rich imagination, and numerous perceptions are the main manifestations of VR technology. Immersion means that any type of computer can build a virtual world based on VR technology. Human-computer interaction indicates that participating users can get corresponding feedback in the virtual world [7, 8]. Through the effective simulation of the natural world, VR technology can provide sufficient imagination environment and space for participating users [9]. Therefore, the application of VR technology in classroom teaching can enrich teachers' teaching means, create a virtual environment for students, mobilize students' enthusiasm, give play to students' imagination, improve learning efficiency, and reduce teachers' teaching intensity. It can also bring multiple senses of vision, hearing, taste, and touch to the students participating in the classroom and improve their learning experience [10].

VR is a new multimedia technology, and experts and scholars have explored VR applications in ordinary classroom teaching. Mahaffey (2018) combined web-based learning, virtual laboratory, tactile learning, and face-to-face human psychology teaching. They reported that this teaching mode significantly improved the information retention rate of students completing online homework; simultaneously, the virtual laboratory was considered safe and effective. These results were significant for students' computer adaptability tests and evaluations [11]. Daineko et al. (2017) introduced the virtual laboratory concept into teaching. They believed that introducing modern information and computer technology could enhance teaching experience and quality [12]. Chilton et al. (2019) integrated the innovative teaching strategy based on virtual teaching into the online theory course of nursing undergraduates to help students understand the research process and transform nursing theories into practice. The simulation found that teaching research processing based on the virtual environment provided an effective educational strategy [13]. Although many studies have studied virtual teaching, there is little research on the application of VR technology in preschool physical education in colleges and universities.

Therefore, this paper discusses the application of VR technology in preschool physical education in colleges and universities by referring to previous literature. A questionnaire survey is conducted on the development of virtual teaching in the sports dance curriculum in the preschool education major direction at two college and universities in Xi'an, Shaanxi, and Zhengzhou, Henan. Then, the samples are divided into an experimental group and a control group to compare the statistical results of preschool sports dance teaching before and after applying VR technology. Compared with traditional teaching methods, VR technology can show the charm of modern teaching and meet the standards of modern digital and information education. Teaching activities need the help of specific technical means, and specialized teaching means need to be continuously improved and reformed to improve the thinking of teachers and students continuously. Therefore, traditional teaching should also use VR technology to break through conventional thinking and bring innovative and breakthrough virtual teaching modes to traditional education. The innovation of this paper lies in optimizing the teaching mode by VR technology and verifying the feasibility of the technology by practical testing.

## 2. Optimization of the Teaching Mode of Preschool Physical Education Based on VR Technology

### 2.1. Theoretical Basis of the Application of VR Technology to the Teaching of Sports Dance in the Preschool Education Major in Colleges and Universities

#### 2.1.1. Behaviorism Learning Theory

The virtual teaching model that combines VR technology with teaching activities is similar to distance teaching. Behaviorism theory holds that learning is a combination of stimulation and response. Behaviorism mainly studies the interactive relationship between environment and behavior and adopts empirical research methods such as experiment, observation, test, and quantification to form a standardized social behavior research paradigm [14, 15]. The basic assumption is that behavior is the learner's response to environmental stimuli. The virtual teaching process can be regarded as a particular response of teachers and students' sensory organs stimulated by VR technology and teaching information. There is a correlation between knowledge and response. In teaching practice, behaviorism learning theory requires teachers to create a learning environment in teaching activities, grasp students' behavior, strengthen students' appropriate behavior, and eliminate inappropriate behavior [16]. In addition, behaviorist learning theory advocates constantly enhancing the relationship between the teaching information of VR technology and the behavior response of learning subjects to realize the teaching behavior of distance education under the isolation of teachers and students. According to this theory, teachers must start with the problems existing in students (behavior deviation), design and study a series of schemes to solve existing problems (strengthen stimulation and change behavior), test the causal relationship between the system and the problem, and finally repeat the test, collect data, and draw a conclusion [17]. Therefore, the behaviorism theory proves the effectiveness of applying VR technology to classroom teaching.

#### 2.1.2. Cognitive Theory

Cognitive theory is a variety of psychological theories about the internal processing process of learners' learning, such as the acquisition and memory of information, knowledge, and experience, achieving epiphany, connecting ideas and concepts, and solving problems. The cognitive theory holds that organisms acquire perceptual or mental structure formation and change. In other words, the foundation of learning is the formation and reorganization of the internal and organized structure of the organism. The principal variables affecting study are the integrity of stimulating situations, sudden understanding or perception, meaningful discovery and acceptance, the characteristics of cognitive structure, and attention or aspiration (higher cognitive learning). VR technology has a dual impact on cognitive ability because of its technical features. The positive effect can improve subjects' cognitive ability, expand the scope of mental objects, and enhance the relationship between subjects and objects [18, 19]. Without complete standards and norms, there will inevitably be a debate between virtual and reality regarding negative impact. In the teaching process, students are active explorers; teachers should create a situation that allows students to independently think, explore the context of the inquiry, and cultivate students' learning ability rather than provide ready-made knowledge [20]. Therefore, VR technology is applied to preschool physical education major direction. First, cognitive psychological activities of learners are analyzed and understood to improve learners' attention and experience, enable learners to assimilate new and old knowledge faster and more efficiently, and establish their reasonable cognitive structure. In this way, learners can improve their learning speed and efficiency and develop themselves.

#### 2.1.3. Constructivist Learning Theory

Constructivist learning theory holds that learners can acquire knowledge with the help of others (such as teachers, learning partners, etc.) instead of directly from teachers; meanwhile, they can use the necessary materials to obtain knowledge through meaning construction. Besides, learning is learner-centered under the guidance of teachers. During the process of acquiring new knowledge, the final learning way for learners is actively constructed by themselves according to their previous background knowledge and subject-related experience under the knowledge background given by teachers [21–23].

Here, VR technology is introduced into the teaching activities of the preschool physical education major. The purpose of this investigation is to increase learners' sense of immersion, bring them a combination of sensory stimuli, deepen learners' knowledge mastery, and improve students' learning effect and interest.

### 2.2. Research Methods

The literature research method, expert interview, and questionnaire survey [24] are selected as research methods to extensively explore VR applications in virtual preschool education in colleges and universities.

#### 2.2.1. Literature Research Method

The keyword-based retrieval is conducted for sports dance teaching in colleges and universities, such as sports dance, teaching model, and VR technology, in the International Philosophy and Social Science Literature Library. The literature research method is used to understand the knowledge of physical education and VR applications of physical education, laying a solid foundation for the following teaching experiment [25].

#### 2.2.2. Expert Interview

With VR technology as the theme, face-to-face or telephone interviews were conducted with sports dance coaches and preschool sports experts from two college and universities in Shaanxi and Henan. Meanwhile, an analysis is performed on the impact of VR technology on the teaching of preschool physical education technology and the practical VR applications in colleges and universities. Experts' opinions and suggestions are carefully adopted during the entire interview, providing a profound, diverse, and valuable viewpoint for the following teaching experiment. Specifically, the interview involves basic information, such as educational background, job title, professional level, the application of modern teaching methods in venues and equipment during physical education, and the attitudes toward modern teaching methods [26, 27].  Table 1 provides the basic information of experts obtained through the interview.

#### 2.2.3. Questionnaire Survey

The questionnaires for experts and students are designed, respectively, according to the research needs. Besides, relevant professionals' opinions are asked to modify and improve the questionnaire content. Finally, the questionnaire is determined. Before the formal test, a questionnaire survey is conducted on the VR application for teaching. The questionnaires are issued on the network. Then, a questionnaire survey is performed on the students in the experimental group. On February 25, 2021, the researcher shall reasonably inform the respondents before the investigation.

### 2.3. Questionnaire Survey

The questionnaire aims to test virtual teaching's effect on facilitating teaching and learning. The questionnaire survey focuses on four aspects: the immersion experience, learning interest of students, learning efficiency of students, and teachers' teaching experience. Between March 2021 and June 2021, this questionnaire survey randomly selected students majoring in preschool education in a two college and universities in Xi an, Shaanxi, and Zhengzhou, Henan, as the research object. Moreover, 509 copies of the questionnaire are distributed, 483 valid questionnaires are returned, and the effective rate is close to 94.8%. In addition, the investigators also communicated with the students on the spot to understand the students' evaluation and doubts about the questionnaire and obtain the students' information from multiple angles, ensuring that the questionnaire can genuinely reflect the actual situation of the students. Students can virtually receive many difficult and adventure training experiences (such as those in medical and military practice). This can reduce training costs and the potential risks in the actual situation [28]. Additionally, students can visit places and simulate conditions they cannot reach to enrich their learning experience. Students who enroll in the elective course of sports dance in colleges and universities are taken as research objects in the teaching experiment. The enrolled students are divided into an experimental group and a control group by random sampling, with 20 students in each group. Compared with the control group, the experimental group utilizes VR technology as an additional tool for the traditional teaching system of sports dance. Based on the traditional teaching of sports dance, the curriculum and instruction of the experimental group emphasize students' immersive experience, learning interest, and learning efficiency. In addition, the application effects of VR technology on sports dance teaching are evaluated [29]. This teaching experiment includes the preliminary investigation and the formal test. In both stages of the experiment, two sports dance teaching hours are set up, which lasts 90 minutes and has a resting time of 10 minutes per class. The experiment site is indoor sports dance classrooms in the researched university. An associate professor and two lecturers constitute the grader group.

This experiment uses event-group training as an example of sports dance. In the preliminary investigation, the VR applications are added according to traditional teaching. The focus is on physical strength. Students are instructed to wear VR glasses to watch event-group training teaching videos. Then, students are asked to discuss and practice in pairs. Finally, the teachers explain the critical knowledge, answer and summarize the questions raised by students, revise and improve the VR animations, and highlight the details. After the curriculum, students' attitudes toward the VR applications are surveyed via a questionnaire, as mentioned in the earlier design.

### 2.4. Experimental Design

The research students are divided into the experimental and control groups through random sampling, with 20 people in each group. VR applications are introduced into the traditional preschool sports dance teaching system as an auxiliary means for the experimental group. Teaching for the experimental group pays more attention to the immersive experience, students' interest, and efficiency based on traditional preschool sports dance teaching to evaluate the teaching effect of VR technology. This experiment mainly includes two parts, i.e., preliminary investigation and formal test. Both parts of the experiment set up two preschool sports dance teaching classes. Before the experiment begins, all students need to take a predictive test to confirm that their overall physical fitness and skills are at the same level.  Table 2 illustrates the basic situation of this formal teaching experiment and physical education curriculum requirements and evaluation indicators.

In addition, the statistical analysis is carried out on the questionnaire results of the predictive test. The students' physical fitness indicators and scoring results before and after the experiment are compared to examine the impact of VR technology and virtual teaching on the teaching model of preschool physical education in colleges and universities. Then, countermeasures and suggestions are proposed for physical education in colleges and universities accordingly. In the analysis of the results, V represents “very understand” or “very satisfied”; F denotes “understand” or “satisfied”; G means “generally understand” or “generally satisfied”; D indicates “do not understand” or “dissatisfied”; N refers to “extremely incomprehensible” or “very dissatisfied.” The following hypotheses are proposed based on the above survey:

1. Virtual teaching can improve students' learning interests to a certain extent
2. Virtual teaching can accelerate students' learning progress to a certain extent
3. Virtual teaching can improve students' learning efficiency to some extent
4. Virtual teaching can reduce teachers' workload to a certain extent

## 3. Results

### 3.1. Questionnaire Results Based on VR Applications

**Figure 1:**
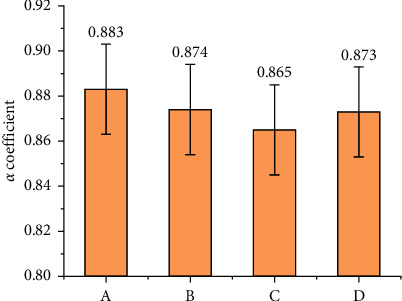
Results of reliability analysis (A: students' immersive experience; B: learning interest; C: learning efficiency; D: teachers' teaching experience).

Figure 1 and Table 3 illustrate the reliability and validity of the questionnaire.

In Figure 1, *α* coefficients of each dimension are more than 0.8, indicating that the overall internal reliability of the questionnaire is excellent. Table 3 reveals the validity analysis results of the questionnaire.

**Figure 2:**
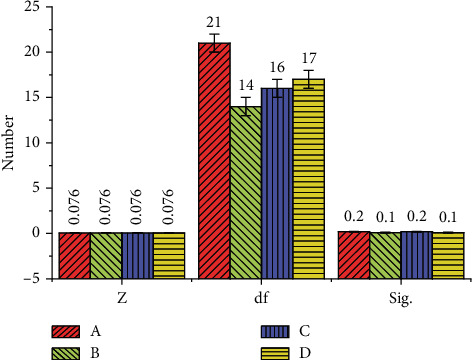
Statistical results of the questionnaire (A: students' immersion experience; B: learning interest; C: learning efficiency; D teachers' teaching experience; *Z* denotes statistics; Sig represents the significance; df is the degree of freedom).

In Figure 1, *α* coefficients of the four variables are higher than 0.8, indicating that the overall internal reliability of the questionnaire is excellent. Table 3 shows that the measurement contents of each variable are both relevant and independent, demonstrating that the questionnaire has outstanding structural validity.  Figure 2 reveals the questionnaire results.

**Figure 3:**
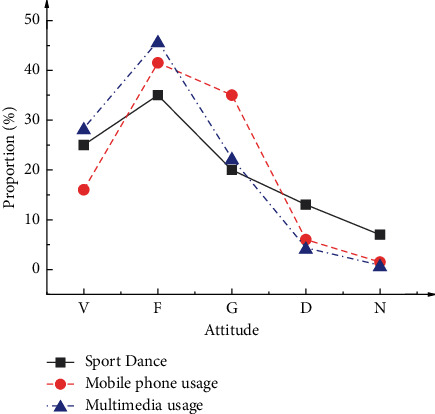
Survey results based on sports dance and modern teaching methods (V represents “very understanding” or “very satisfied”; F denotes “understand” or “satisfied”; G means “generally understand” or “generally satisfied”; D indicates “do not understand” or “dissatisfied”; N refers to “extremely incomprehensible” or “very dissatisfied”).

According to Figure 2, the Sig. values of four dimensions are all higher than 0.05 and obey normal distribution.  Figure 3 shows the survey results of participants' understanding and mastery of sports dance lessons and their attitudes toward VR applications during the class.

Figure 3 suggests that 90.0% of survey participants contact and learn sports dance, which corroborates the theme of this experiment and further confirms the effectiveness of this questionnaire. Most survey participants have some experience in sports dance lessons and have a clearer understanding of their overall learning levels. Besides, 57.55% of participants favor using mobile phones, while only 7.50% disagree with using mobile phones as an auxiliary tool for physical education teaching. In addition, 73.51% of participants agree with using multimedia devices, while only 4.50% disagree. The reason is the convenience of modern technologies, such as mobile phones and multimedia devices, in sharing and studying the learning materials.

**Figure 4:**
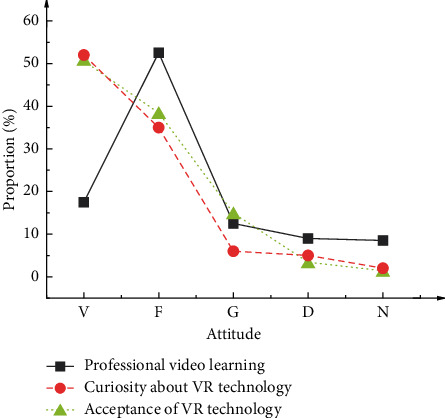
Survey results based on VR applications for sports dance teaching of the preschool education major direction (V represents “very understanding” or “very satisfied”; F denotes “understand” or “satisfied”; G means “generally understand” or “generally satisfied”; D indicates “do not understand” or “dissatisfied”; N refers to “extremely incomprehensible” or “very dissatisfied”).

Figure 4 illustrates the survey results based on VR applications in preschool physical education major directions' dance teaching classrooms in colleges and universities.

Figure 4 demonstrates that 70.0% of participants watch professional event-group training videos frequently, suggesting that nearly two-thirds of participants improve their abilities by watching professional sports dance videos. Such a result is closely correlated with participants' major direction and new technologies, such as network communication. Furthermore, 88.0% of participants are highly curious about using VR technology, and 88.6% of those accept VR applications in sports dance teaching. Hence, VR technology is trendy in preschool physical education major's sports dance teaching of colleges and universities.

**Figure 5:**
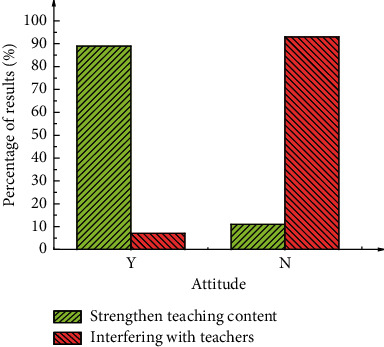
Attitudes of survey participants toward VR technology during teaching.

Figure 5 shows the survey results of the participants' attitudes to VR technology during teaching.

Figure 5 illustrates that 89.1% of survey participants believe that VR technology can further students' understanding of the teaching contents. In addition, 93.0% of survey participants think that VR technology does not interfere with the teachers' illustrations.

**Figure 6:**
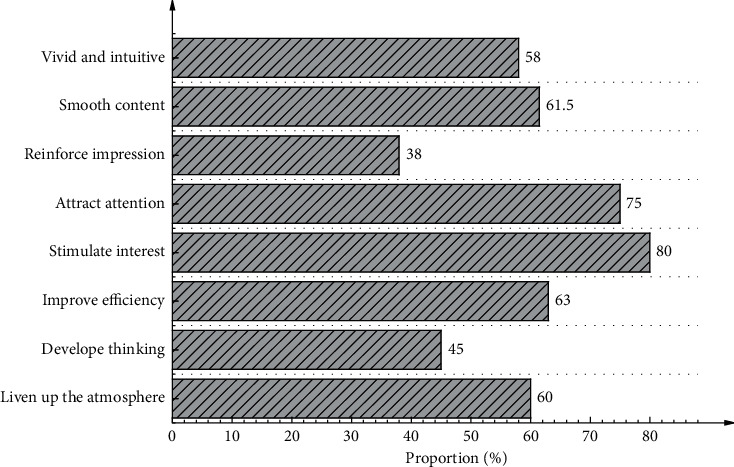
Advantages of virtual physical education classes.

Figure 6 describes the advantages of virtual teaching for physical education compared with traditional physical education teaching.

Figure 6 demonstrates that the proportion of participants that believe virtual teaching can stimulate students' learning interest is the highest, reaching 80%, followed by those who opine that virtual teaching could attract students' attention, accounting for 75%. In addition, the proportion of participants that claim that virtual teaching can improve learning efficiency reaches 63%. Overall, these findings comprehensively indicate the characteristics of VR technology.

### 3.2. Questionnaire Results Based on Preliminary Investigation

**Figure 7:**
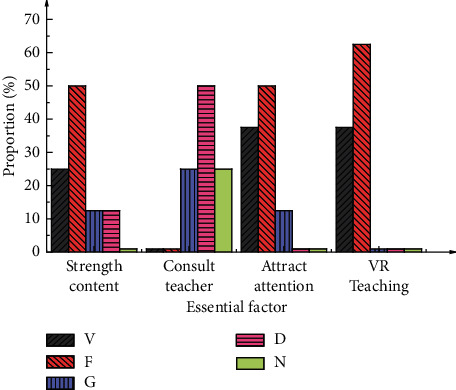
Questionnaire results based on the preliminary investigation.

Figure 7 shows the questionnaire results in the preliminary investigation.

Figure 7 shows that, after VR technology is introduced into the sports dance course, the participants have a deeper understanding, increase learning efficiency, and significantly accelerate learning progress. Besides, participants become more concentrated, and their dependence on teachers decreases, reducing teachers' workload. These results demonstrate that the participants are optimistic and have higher expectations about applying VR technology to sports dance teaching.

### 3.3. Statistical Results Based on Formal Test

Before the formal test, the physical fitness indicators of students in both groups are counted, as shown in Table 4.

The *t*-test results of the students' physical fitness in each group reveal that the significance level *P* corresponding to each primary indicator is higher than 0.05, suggesting that the basic data onto physical fitness between the two groups are not significantly different. Thus, the control variables based on this experiment are consistent.

**Figure 8:**
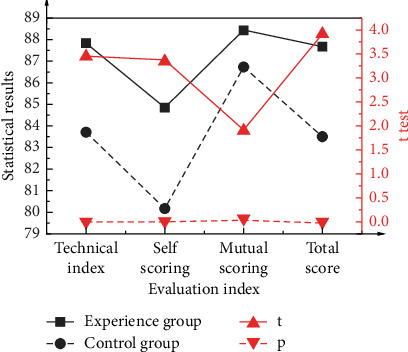
Performance comparison between the experimental group and the control group.

Figure 8 shows the final performance comparison between the experimental and control groups in the formal test.

**Figure 9:**
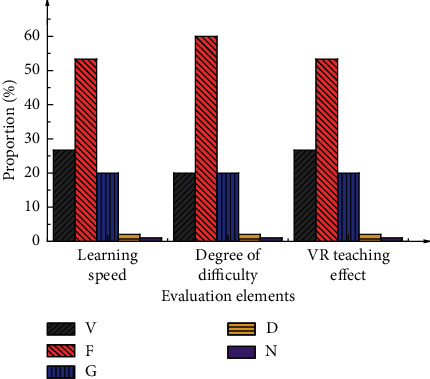
Statistical results of VR applications based on the experimental group.

According to the statistics in Figure 8, the experimental group's performance is slightly better than that of the control group, and the average score of the experimental group is 4.13 points higher than that of the control group. Meanwhile, the corresponding *P*-value is 0.003 (*P* < 0.05), proving a significant difference between the two groups. In terms of self-evaluation scores, the average score of the experimental group is 4.68 points higher than that of the control group, and the corresponding *P*-value is 0.012 (*P* < 0.05), showing a significant difference. In terms of mutual evaluation scores, the average score of the experimental group is 1.70 points higher than that of the control group, and the corresponding *P*-value is 0.067, greater than 0.05, so there is no significant difference. Overall, the average score of the experimental group is 3.18 points higher than that of the control group, and the corresponding *P*-value is 0.001 (*P* < 0.05), showing a significant difference. The statistical analysis of experimental data verifies Hypothesis 1 and Hypothesis 3.  Figure 9 shows the statistical results of students' attitudes toward VR applications based on the experimental group in the formal test.

According to Figure 9, the students who participate in the questionnaire agree that introducing VR technology into teaching can deepen students' understanding, improve students' interest in learning, and deepen students' immersion experience. However, 80.0% of the participants believe that virtual teaching has positively promoted preschool physical education's teaching mode and curriculum design in colleges and universities. Hypothesis 2 and Hypothesis 4 are validated through statistical analysis of experimental data.

## 4. Discussion

To sum up, the formal test results verify the effectiveness of virtual teaching in preschool physical education in colleges. The comparative analysis of technical indicators and self-evaluation indicators shows that VR technology has an evident impact on physical training and teaching in colleges. The experimental results indicate that virtual teaching can improve students' technical level from the technical indicators. In addition, VR technology enables students to understand preschool physical education content. Consequently, students are more satisfied with their academic performance, and their self-confidence and interest in learning can be improved simultaneously. Compared with the control group, the experimental group has higher learning efficiency, enhanced immersion, and a deeper understanding of textbooks. Compared with similar studies, the results of this paper prove that the introduction of new technologies in the classroom can improve the teaching effect of the classroom and effectively enhance the students' class experience.

The survey results suggest that most participants are very interested in VR technology and agree to apply VR technology to physical training teaching. In addition, most participants believe that the introduction of VR technology can enhance students' understanding of teaching content and reduce interference to teachers. Overall, participants agree with the teaching mode of virtual sports dance and the corresponding curriculum. VR technology also improves students' interest in learning, effectively increases students' attention in class, and increases students' class experience with a vivid classroom teaching model. The preliminary investigation confirms the participants' attitude toward the sports virtual teaching mode and validates the questionnaire survey results. In addition, questionnaires and teaching experiments show that virtual teaching based on VR technology can provide learners with a more authentic and intuitive experience. VR technology also saves teaching resources while providing an immersive effect. Therefore, the whole learning process is very convenient, and the workload of students and teachers is reduced. Therefore, VR technology can provide novel and practical development ideas for transforming teaching mode and innovation of curriculum setting of preschool education major direction in colleges and universities.

## 5. Conclusions

Literature research, expert interview, and questionnaire survey are adopted to analyze the impact of VR technology on physical training teaching in a college to study the application effect of VR technology in the classroom. The experimental results show that all the research hypotheses are valid. The *t*-test results of the two groups of students demonstrate that the primary data of the two groups have no significant difference in physique. At the same time, participants believed that the introduction of VR technology could deepen students' understanding, improve students' interest in learning, and deepen students' sense of immersion. Specifically, 80.0% thought that virtual teaching could stimulate students' interest in learning, 75.0% said that VR application could attract students' attention, and 63.0% thought that VR application could improve learning efficiency. This study has promoted the teaching mode and curriculum design of preschool physical education training in colleges and universities. In addition, participants have a deeper understanding of physical training, improve learning efficiency, and significantly speed up learning progress. VR technology makes students more concentrated, reduces their dependence on teachers, and reduces students' academic burden. This also shows that the students are optimistic about the application of VR technology in sports dance teaching.

However, there are some deficiencies in the current research. For example, students' participation is low, and the questionnaire survey has not been paid attention to by teachers and students, resulting in insufficient data. Moreover, the application mode of VR technology in the classroom is in its infancy, which is insufficient to furnish students with a perfect class experience. Therefore, future research will expand the survey population of the questionnaire and consider the specific situation of physical education teaching in all grades to ensure the accuracy of the research conclusions and improve the application effect of VR technology in the classroom.

## Figures and Tables

**Table 1:** Basic information of experts based on the expert interview method.

Information element		Number of people	Proportion (%)
Educational background	Doctor degree	6	33.3
	Master degree	12	66.7

Job title	Professor	5	27.8
	Associate professor	6	33.3
	Lecturer	6	33.3
	Teaching assistant	1	5.6

Referee certificate	International certificate	5	27.8
	Chinese national certificate	13	72.2

**Table 2:** Basic information and requirements for participants based on the teaching experiment.

Groups	The control group	The experimental group
Gender ratio	Female-100%	Female-100%
Number of people	20	20
Average age (years)	21	21
Teaching requirements and curriculum	Before the class: students watch 2D instructional videos and textbooks per the content released by the teacher as preview During the class: the teacher gives lessons as usual After the course: students watch 2D instructional videos and textbooks per the content released by the teacher as a review	Before the course: students watch virtual teaching videos released by the teacher wearing VR glasses During the course: the teacher gives lessons like before Before the after-class: students watch virtual teaching videos released by the teacher wearing VR glasses as a review
Physical fitness indicators	Sit-ups; straddle jumps; balance on one foot with eyes closed; horizontal twist	
Technical indicators	Pose and balance; action quality	

**Table 3:** Results of validity analysis.

	A	B	C	D
A	1			
B	0.553^∗∗^	1		
C	0.567^∗∗^	0.632^∗∗^	1	
D	0.523^∗∗^	0.651^∗∗^	0.673^∗∗^	1

^∗∗^The correlation is significant at the 0.1 level (A: students' immersion experience; B: learning interest; C: learning efficiency; D: teachers' teaching experience).

**Table 4:** Basic statistics of physical fitness indicators of students in both groups.

Groups	Physical fitness indicators			
	Sit-ups in 1 min	Straddle jumps in 15 s	Balance on one leg with eyes closed	Horizontal twist
The experimental group	27.92 ± 8.25	3.41 ± 1.50	30.66 ± 14.52	3.32 ± 1.24
The control group	29.22 ± 7.32	2.48 ± 1.25	35.00 ± 14.72	2.60 ± 1.17
*t*	−0.443	1.925	−0.815	1.662
*P*	0.662	0.065	0.424	0.109

## Data Availability

The data used to support the findings of this study are included within this article.
